# Multivariable modelling of factors associated with criminal convictions among people experiencing homelessness and serious mental illness: a multi-year study

**DOI:** 10.1038/s41598-021-96186-x

**Published:** 2021-08-16

**Authors:** Milad Parpouchi, Akm Moniruzzaman, Jane A. Buxton, Julian M. Somers

**Affiliations:** 1 Somers Research Group, Faculty of Health Sciences, Simon Fraser University, Blusson Hall, Room 11830 – 8888 University Dr., Burnaby, BC V5A 1S6 Canada; 2 School of Population and Public Health, Faculty of Medicine, University of British Columbia, 2206 East Mall, Vancouver, BC V6Z 1Z3 Canada; 3 British Columbia Centre for Disease Control, 655 West 12th Avenue, Vancouver, BC V5Z 4R4 Canada

**Keywords:** Epidemiology, Risk factors

## Abstract

People experiencing homelessness and serious mental illness exhibit high rates of criminal justice system involvement. Researchers have debated the causes of such involvement among people experiencing serious mental illness, including what services to prioritize. Some, for example, have emphasized mental illness while others have emphasized poverty. We examined factors associated with criminal convictions among people experiencing homelessness and serious mental illness recruited to the Vancouver At Home study. Participants were recruited between October 2009 and June 2011. Comprehensive administrative data were examined over the five-year period preceding study baseline to identify risk and protective factors associated with criminal convictions among participants (n = 425). Eight variables were independently associated with criminal convictions, some of which included drug dependence (RR = 1.53; *P* = 0.009), psychiatric hospitalization (RR = 1.44; *P* = 0.030), an irregular frequency of social assistance payments (compared to regular payments; 1.75; *P* < 0.001), and prior conviction (RR = 3.56; *P* < 0.001). Collectively, findings of the present study implicate poverty, social marginalization, crises involving mental illness, and the need for long-term recovery-oriented services that address these conditions to reduce criminal convictions among people experiencing homelessness and serious mental illness.

## Introduction

Prior studies have consistently reported disproportionate criminal justice system involvement (CJSI) among people experiencing homelessness ^e.g.,^^1–4^ or serious mental illness ^e.g.,^^5–7^. People experiencing both homelessness and serious mental illness may have even higher rates of CJSI compared to people experiencing serious mental illness only^8^. However, the relative contributions of homelessness and serious mental illness to the risk of CJSI among people who experience both is less clear^8^.

Homelessness itself has been found to be independently associated with CJSI^9–11^, as the behaviour of people living on the streets is more visible^12^. Laws may also prohibit behaviours inherent to homelessness, such as sleeping on the streets^13^. Moreover, people experiencing homelessness, including those with mental illness, are more likely to be arrested for or to commit crimes of a more minor or non-violent nature, which may be related to visibility as well as survival and subsistence^1,2,14–16^. Mental health symptoms have also been found to predict committing non-violent crimes among PEHSMI^14^. It is important to note, however, that PEHSMI have also been found to commit major crimes at rates higher than the general population^12^, and serious mental illnesses have been found to be associated with a higher risk of violent crime^17,18^.

There is widespread agreement that disinvestments in and inadequate provision of supports and services are responsible for the overrepresentation of people experiencing serious mental illness in the criminal justice system. However, the role of mental illness itself and what specific services to prioritize to reduce CJSI have been the subject of debate^e.g.,^^19–21^. A hypothesis known as “the criminalization of mentally disordered behavior” dating back to the 1970s causally links deinstitutionalization from psychiatric hospitals with a consequent increase in CJSI of people experiencing serious mental illness^22^. Lamb and Weinberger described this phenomenon as having developed because of “the failure of the mental health system to provide a sufficient range of treatment interventions, including an adequate number of psychiatric inpatient beds”^23^. Although they recognize the importance of community-based treatment and psychosocial supports, Lamb and Weinberger^23^ further argue that “there is a substantial minority who need the structure and support of acute, intermediate, or long-term care in a hospital setting or a highly structured, locked 24-hour care community facility [and that this] …is absolutely essential if deinstitutionalization and the reduction of criminalization are to be successful”. Some researchers have found mental health symptoms to be associated with CJSI^14,24^. Other researchers have found that mental health symptoms and subsistence-related crimes are not the primary reason for disproportionate CJSI among people experiencing serious mental illness and instead criminogenic traits (e.g., impulsivity) irrespective of serious mental illness are more primary drivers, the treatment of which may have greater effect^21^. Still, other researchers have argued that increasing the number of psychiatric hospital beds would have little impact on CJSI and that for most people living with serious mental illness:

“…the key to staying out of hospitals, jails, and prisons may be a place to live, a job or some income support, a meaningful relationship or social network, quality healthcare, or linkage to treatment instead of frequent arrest for substance use disorders-fundamental needs that can best be redressed in the community, not psychiatric or correctional institutions”^19^.

In describing adverse social outcomes among people living with serious mental illness, such as CJSI and homelessness, Draine et al.^20^ argue “…that mental illness is not as potent an explanatory factor for these problems as the psychiatric literature might lead us to believe” and that the social context is key in that the relationship between serious mental illness and adverse social outcomes, such as CJSI, are strongly moderated by poverty-related factors (e.g., low income, substance use, unemployment, etc.).

An alternative to the criminalization of mentally disordered behaviour hypothesis is the General Personality and Cognitive Social Learning model^25^, positing “that the causes of crime are to be found within the individual and his/her social learning environment”^26^. According to this model, there are a number of causal factors that determine criminal conduct, and these factors can be grouped in terms of their strength of association with crime (minor vs. moderate vs. major predictors). Serious mental disorders and their symptoms are considered minor risk factors at most, along with the following other minor risk factors: neighborhood, age, ethnicity, gender, family of origin, and temperament/mental health. Moderate risk factors include the following variables grouped as the “Moderate Four”: education/employment, family/marital, substance abuse, and leisure/recreation. These risk factors are posited as contextualizing and having implications for rewards/reinforcement and punishments as they relate to crime. An example is provided where if one is unemployed then incarceration may entail less punishment compared to one who is employed and may hence lose their job. Employment may also provide an environment with reinforcers of prosocial behaviour. The major risk factors are grouped as the “Big Four” and include: criminal history, antisocial associates, antisocial personality pattern, and antisocial cognition. Andrews and Bonta combined the Moderate Four and the Big Four calling it the Central Eight^25,26^. In a comprehensive international meta-analysis, Bonta et al.^26^ found that all factors of the Central Eight significantly predicted recidivism (any offence), as measured by arrests and convictions, among people experiencing mental illness; predictors with the largest effect sizes included substance abuse, procriminal attitudes and cognitions, and antisocial personality pattern (which included antisocial personality disorder). Interestingly, other than personality disorder, and antisocial personality disorder in particular, none of the mental illness-related variables were found to be significantly associated with recidivism. An earlier meta-analysis included five of the Central Eight risk factors in analyses and found four to be significantly associated with recidivism, including adult criminal history, antisocial personality disorder, family problems/single marital status, and substance abuse; education and employment were not significantly associated with recidivism. Having a mental disorder was found to be protective against recidivism while previous psychiatric hospitalizations increased the risk^27^.

Correctly identifying factors associated with CJSI is important for informing relevant public policies and services to reduce CJSI. Unresolved questions about the factors contributing to the disproportionate CJSI of people experiencing serious mental illness have fundamental implications for interventions, including what services and supports to prioritize.

Specifically among PEHSMI, factors that have been found to be associated with CJSI have not been investigated in multivariable models that also use objective sources of data over long periods of time. Based on a systematic review of CJSI among PEHSMI^8^, most previous studies examining correlates have been conducted using self-reported data, and those with longitudinal data have spanned periods of less than 2 years. Moreover, the majority have been based in the U.S. To the best of our knowledge, no subsequent studies have addressed all of these limitations.

Use of administrative data as a measure of service use is commonly regarded as a “gold standard” approach^28^ and overcomes limitations associated with bias including decayed accuracy of recall. Although agreement between self-reported and administrative CJSI data has been reported as “good”^28^ or “substantial”^29^ among PEHSMI, these are not the highest levels of agreement, and it has been found that under-reporting is a potential problem when specifically asking about the number of occurrences of CJSI as opposed to any occurrence^28^ as well as when asking about jail^29^. To our knowledge, no prior studies of CJSI among PEHSMI have conducted multivariable analyses that also draw on objective measures for periods of time exceeding two years. To address these limitations, we investigated the CJSI of PEHSMI based on up to 10 years of observation using a Canadian provincial inter-ministry database covering the entire Province of British Columbia (BC), with linked comprehensive justice, health, and social services-related data. Our primary objective was to identify risk and protective factors associated with criminal convictions over a five-year period using multivariable modelling.

## Methods

### Study overview, data sources, participant recruitment, and procedures

**Figure 1:**
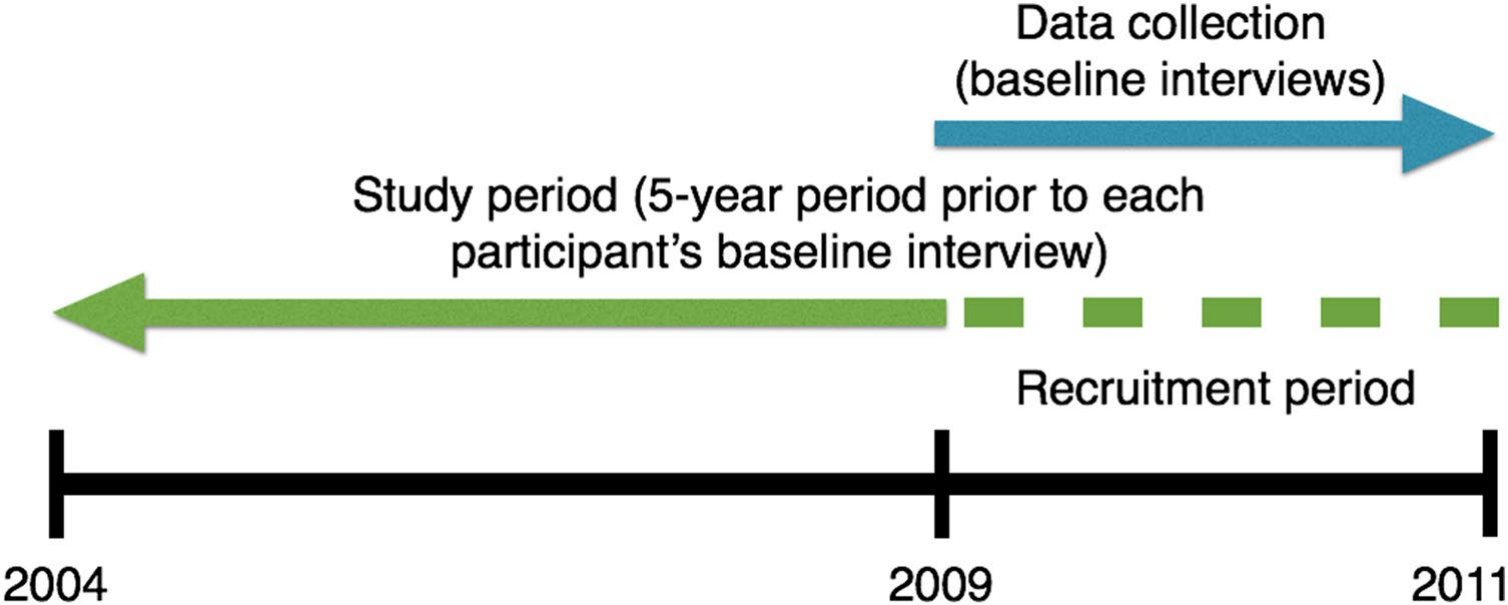
Study timeline. The green dashed line signifies the period during which participants were recruited and which in turn defines the end point of the five-year pre-baseline study period.

Data were collected from a larger experiment. Participants were recruited between October 2009 and June 2011 and completed a baseline interview during this period. For the present analyses, in addition to self-reported data collected at the baseline interview, administrative data from participants were examined retrospectively and included the five-year period preceding their baseline interview. Figure 1 presents the study period in relation to the data collection period.

The data source for the present analyses was the Vancouver At Home (VAH) study^30^. VAH was mounted to investigate the effects of supported housing, specifically Housing First, on a variety of outcomes among PEHSMI in Vancouver, BC, Canada. Although two randomized controlled trials were included in VAH (Current Controlled Trials: ISRCTN57595077 and ISRCTN66721740), the current study only analyzed pre-randomization data and included participants from both trials. Study procedures adhered to relevant guidelines and regulations. The Research Ethics Board of Simon Fraser University approved the study.

Community agencies and institutions providing services to PEHSMI (e.g., drop-in centres) located throughout Metro Vancouver referred potential participants to VAH from October 2009 and June 2011. Eligibility screening involved two steps: (1) briefly over the phone with the referring agency, and for those seemingly eligible (2) more comprehensive in-person screening. Referred individuals were enrolled in the study if they met eligibility criteria. These criteria included: (1) Canadian citizenship, (2) age 19 or older, (3) absolutely homeless or unstably housed, and (4) serious mental illness according to the Mini International Neuropsychiatric Interview (MINI) criteria^31^. Potential participants were considered absolutely homeless if they had “no fixed place to sleep or live for more than 7 nights [in the past week] and little likelihood of obtaining accommodation in the coming month”^30^. They were considered unstably housed if they resided “in marginal accommodation, such as a SRO [single-room occupancy] hotel, and having two or more episodes of [absolute] homelessness (as defined above) during the past 12 months”^30^. All participants provided written informed consent. Separate informed consent procedures addressed: (1) consent to participate in VAH and (2) consent to access participants’ administrative records from three BC Government ministries (Ministries of Justice, Health, and Social Development and Social Innovation). After enrollment, an in-depth baseline interview was conducted, lasting about 90–180 min. One randomized controlled trial was mounted for participants with a high level of need for support and another for those with a moderate need. A participant was considered as having a high level of need for support if they had a psychotic or bipolar disorder (per MINI), received a score of ≤ 62 on the Multnomah Community Ability Scale^32,33^, and had one or more of: (1) a history of arrest or incarceration in the past six months, (2) two or more psychiatric hospitalizations in one of the past five years, or (3) substance dependence (per MINI) in the past month. Participants who did not meet the high needs inclusion criteria were considered as having a moderate level of need for support. The present analyses included socio-demographic information collected during the baseline interviews.

The Inter-Ministry Research Initiative (IMRI) was utilized to access comprehensive, linked administrative data from the BC Ministries of Justice (data availability: from 1997 to study randomization), Health (data availability: from 1990 to study randomization), and Social Development and Social Innovation (data availability: 1997 to study randomization; currently, this ministry is called the BC Ministry of Social Development and Poverty Reduction)^34^. All provincial conviction-related information was accessed using the Ministry of Justice data included in the IMRI. Anyone at least 18 years of age sentenced in a court in BC is entered into this database. All health service use-related information came from the Ministry of Health data contained in the IMRI. Billing data from the universal health insurance plan in BC, called Medical Services Plan (MSP), comprises a subset of the data from the Ministry of Health and was used to ascertain participant diagnostic information based on The International Classification of Diseases, Ninth Revision (ICD-9) codes. The diagnoses represented by these codes are determined by licensed health professionals in the community for billing purposes. Hospitalization data was included using the Discharge Abstracts Database from the Ministry of Health included in the IMRI. The International Classification of Diseases-10-CA, Canada (ICD-10-CA) was used to determine the disorder related to hospitalization. A list of relevant ICD-9 and ICD-10-CA codes and their descriptions used for the current analyses are presented in Supplementary Tables S1 to S4. Social assistance information in the IMRI came from the Ministry of Social Development and Social Innovation. Additional VAH details regarding participant recruitment, study procedures, and power calculations have been outlined in Somers et al.^30^. Further details about the IMRI have also been published^34^.

### Variables of interest

Variables were included based on peer-reviewed literature and availability of data in VAH and the IMRI. A few of the socio-demographic variables collected during the VAH baseline interview were included as independent variables in the current analyses, including age at randomization and the following which were not expected to change over time: gender, ethnicity, education, and age of first experiencing homelessness. Details concerning questionnaires used in VAH have been published^30^.

Administrative data from the IMRI were used for the remaining independent variables included in the current analyses. Criminal convictions (related to federal and provincial offences) occurring in any court in BC were included. Convictions resulting in incarceration in provincial (< 2 years) or federal prisons (≥ 2 years) were included. The date of the offence leading to conviction (as opposed to the date of conviction) was used in analyses. All offences reported were ones that led to conviction. Offence types were also reported (e.g., drug and alcohol-related, breach of court order, property, or violent). We included the following non-substance use-related mental disorders (NSMD), which were included as part of MSP data using ICD-9 diagnostic codes: schizophrenia (ICD-9 code: 295), bipolar disorder (ICD-9 code: 296), depressive disorder (ICD-9 code: 311) neurotic disorder (ICD-9 code: 300), and personality disorder (ICD-9 code: 301). Substance use disorders were identified in a similar manner and included: alcohol dependence (ICD-9 code: 303), drug dependence (ICD-9 code: 304), and nondependent drug abuse (ICD-9 code: 305). Hospitalization data included the following types: psychiatric (NSMD-related; ICD-10-CA codes: F00-F89 except F10-F19), substance use disorder-related (ICD-10-CA codes: F10-F19), and non-psychiatric (all codes except F00-F89). ICD-9 codes for mental disorders have been used in previous studies^35,36^. Annual frequencies of social assistance payments were also included in the current analyses.

### Statistical analysis

Participants who met criteria for each of the VAH randomized controlled trials were pooled in the current analyses to increase power. The study period consisted of the five-year period immediately preceding VAH baseline. Descriptive analyses were conducted and reported with means and standard deviations for continuous variables and frequencies and percentages for categorical variables. All variables measured using administrative records from the IMRI were reported using their values during the year preceding VAH baseline, while self-reported variables were reported using their values at VAH baseline. Descriptive convicted offence data were reported for the two-years preceding the study period (i.e., years 6 and 7 before baseline) and the ten-year period preceding VAH baseline.

The dependent variable was the number of convicted offences. This variable was measured as a count in each year of the five-year period preceding study baseline. A panel data structure was employed to measure the relationship between all independent variables and the dependent variable. Similar to the dependent variable, all time-varying independent variables were calculated in each year (annualized) of the study period (i.e., each of the 5 years preceding baseline). Data from all five years preceding baseline were included in the present analyses. Generalized estimating equations (GEE), a longitudinal analytic method, were conducted due to the use of repeated measures^37^. GEE specifications involved a negative binomial distribution with a log link function due to the count nature of the outcome data. An exchangeable correlation structure was further specified to address the dependency of within-subject observations over time. Robust standard errors were used to protect against potential mis-specification and heteroskedasticity^38^. Dispersion parameters for the GEE models were imputed using the method suggested by Hilbe^39,40^.

Both bivariate and multivariable GEE was conducted. All variables in the unadjusted model were forced into the adjusted model. A separate multivariable model was also created as a sensitivity analysis including only variables significant at *P* ≤ 0.05 in the bivariate analysis. The following independent variables were treated as fixed: gender (woman/man), ethnicity (Indigenous/White/Other), education (less than high school/high school or more), age of first homelessness (< 25 years/ ≥ 25 years dichotomized based on youth vs. adult), and prior offence (any conviction; during the two-year period preceding the study period). The following independent variables were treated as time-varying and were measured on an annual basis during the five-year study period: age (years), time (years), schizophrenia (yes/no), bipolar disorder (yes/no), depressive disorder (yes/no), neurotic disorder (yes/no), personality disorder (yes/no), alcohol dependence (yes/no), drug dependence (yes/no), nondependent drug abuse (yes/no), psychiatric hospitalization (NSMD-related; yes/no), substance use disorder-related hospitalization (yes/no), non-psychiatric hospitalization (yes/no), and frequency of social assistance payments (none or single/irregular/regular). Controlling for psychiatric hospitalizations may adjust away the effect of the most severe symptoms of serious mental illness. Therefore, a supplementary analysis was also conducted involving a hierarchical GEE negative binomial regression model with three blocks. This was done to examine the relationship between mental disorders and criminal convictions prior to adjustment for related hospitalizations. Only socio-demographic variables were included in the first block. NSMDs and substance use disorders were added for the second block followed by the addition of all hospitalization variables for the third block. Rate ratios, including 95% confidence intervals, were presented as the measure of association. P-values were two-sided with significance set at alpha 0.05. Missing values for self-reported socio-demographic variables at VAH baseline were low (~ 1%) and were replaced with the median value for continuous variables and the largest category for categorical variables. Stata 16^41^ was used to conduct these analyses.

## Results

**Figure 2:**
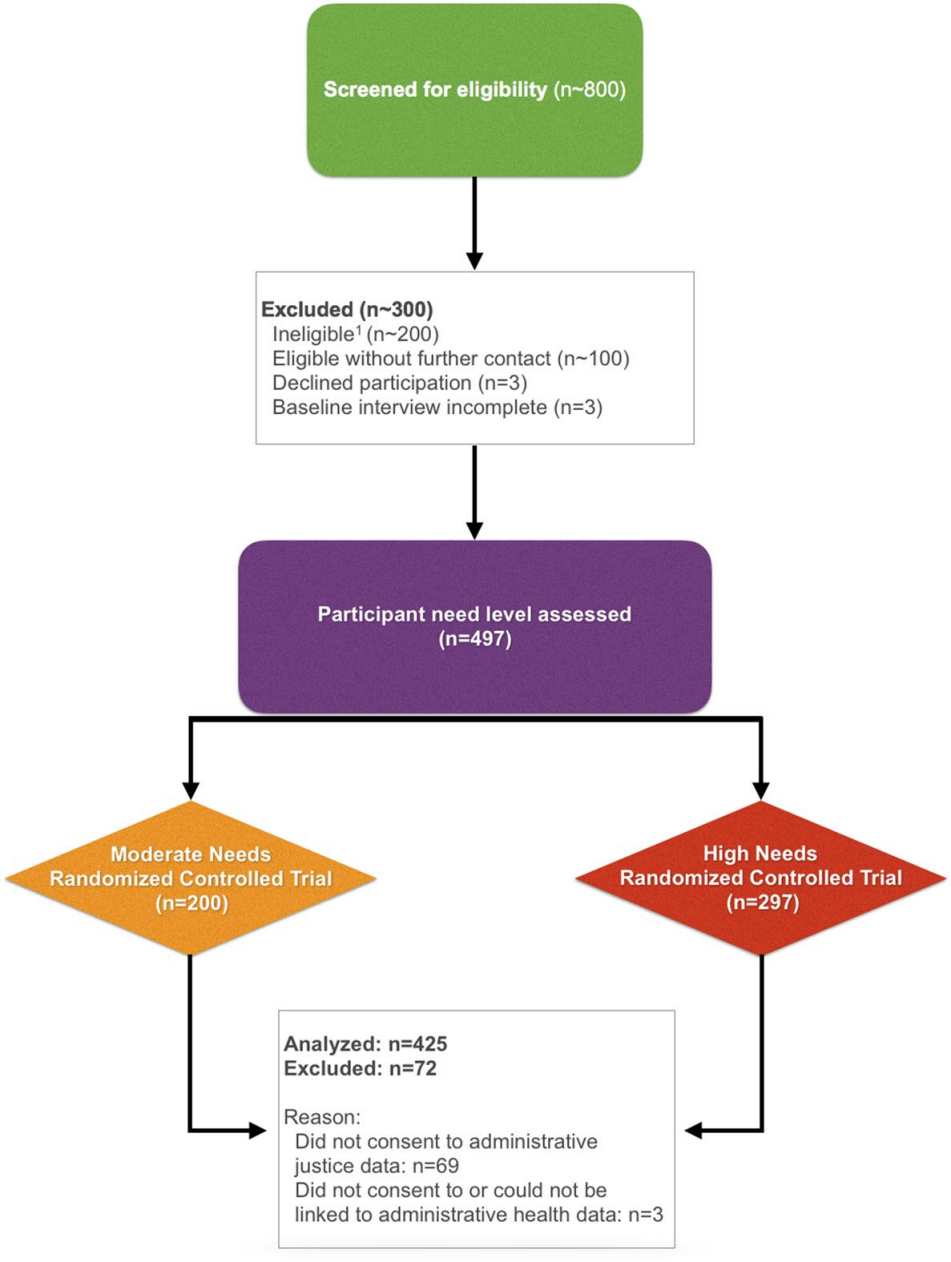
Participant flow-through. ^1^About 100 participants were ineligible after telephone screening, and 94 participants after in-person screening.

A total of 497 participants met inclusion criteria and completed the baseline interview of VAH (200 had moderate needs and 297 had high needs). Of these participants, 425 (85.5%) provided consent to access their administrative data from all three BC ministries and were successfully linked. Prior comparisons between VAH participants who provided consent and those who did not have shown no significant differences^42,43^. A comparison of socio-demographic characteristics (those not expected to change) between VAH participants who did and did not provide consent to access their administrative data is included in Supplementary Table S5. There was a marginally significant difference in ethnicity between groups. Figure 2 displays the flow-through of participants.

Baseline socio-demographic characteristics of participants, as well as social assistance payment frequencies, mental illnesses, substance use disorders, and hospitalizations in the year preceding baseline are presented in Table 1.

**Table 1:** Baseline characteristics of Vancouver At Home participants who consented to administrative data and could be linked (n = 425).

Variable^a^	Mean (SD) / n (%)
**Socio-demographics**	
**Age at randomization (in years)**	
Mean (SD)	40.8 (11.0)
Median (IQR)	41.4 (32.2, 47.8)
< 25 years	33 (7.7)
25–49 years	308 (72.5)
≥ 50 years	84 (19.8)
**Gender, n(%)**	
Woman	107 (25.4)
Man	314 (74.6)
**Ethnicity, n(%)**	
Indigenous	69 (16.2)
White	232 (54.6)
Other	124 (29.2)
**Education level, n(%)**	
High school or higher	178 (42.2)
Less than high school	244 (57.8)
**Age of first homelessness, n(%)**	
< 25 years	187 (44.4)
≥ 25 years	234 (55.6)
**Social assistance payments during the year preceding baseline, n (%)**	
None/single (0–1)	48 (9.4)
Irregular (2–11)	114 (26.8)
Regular (12)	271 (63.8)
**Non-substance-related mental disorders (NSMD) during the year preceding baseline, n(%)**	
Schizophrenia	177 (41.7)
Bipolar disorder	138 (32.5)
Neurotic disorder	108 (25.4)
Personality disorder	36 (8.5)
Depressive disorder	124 (29.2)
**Substance use disorders during the year preceding baseline, n (%)**	
Alcohol dependence	52 (12.2)
Drug dependence	137 (32.2)
Nondependent drug abuse	31 (7.3)
**Acute hospitalization during the year preceding baseline, n (%)**	
Psychiatric (NSMD-related) hospitalization	141 (33.2)
Substance use disorder-related hospitalization	48 (11.3)
Non-psychiatric hospitalization	67 (15.8)

^a^Socio-demographic variables reported at study baseline and all other variables reported using their values in the year before baseline.

Offence-related characteristics are presented in Table 2. The mean number of convicted offences over the five-year study period was 2.95, with an increasing trend every year up to baseline. When examining the ten-year period preceding baseline, the mean number of convicted offences was 5.4. The prevalence of any convicted offence during the five and ten years preceding baseline was 48.5% and 57.7%, respectively. Property offences (mean = 1.3) accounted for nearly half of all offences.

**Table 2:** Convicted offence-related characteristics of Vancouver At Home participants who consented to administrative data and could be linked (n = 425).

Variable	Mean (SD) / n (%)
**Offences during the study period, mean (SD)**	
Year 1/5^th^ last year preceding baseline	0.44 (1.57)
Year 2/4^th^ last year preceding baseline	0.51 (1.46)
Year 3/3^rd^ last year preceding baseline	0.60 (1.53)
Year 4/2^nd^ last year preceding baseline	0.62 (1.46)
Year 5/last year preceding baseline	0.78 (1.72)
Year 1 to year 5 (entire study period)	2.95 (5.6)
**Offences during the study period, n (%)**	
None	219 (51.5)
≥ 1	206 (48.5)
**Offences during the 10-year period preceding baseline, mean (SD)**	5.4 (11.1)
**Offences during the 10-year period preceding baseline, n (%)**	
None	180 (42.3)
≥ 1	245 (57.7)
**Type of offence during the study period, mean (SD)**	
Drug & alcohol-related offence	0.2 (0.7)
Breach offence	0.7 (1.8)
Property offence	1.3 (3.6)
Violent offence	0.6 (1.3)
**Prior offence (any conviction) during the 2-year period preceding study period, n (%)**	
None	332 (78.1)
≥ 1	93 (21.9)

Unadjusted and adjusted rate ratios generated by the bivariate and multivariable GEE analyses are presented in Table 3. Although a number of variables were significantly associated with offending in the bivariate analysis, eight remained significant in the multivariable model, namely age (in years; RR = 0.98; 95% CI 0.96–1.00), time (in years; RR = 1.18; 95% CI 1.08–1.28), being a man (compared to being a woman; RR = 1.49; 95% CI 1.00–2.20), receiving no social assistance payments (compared to receiving them regularly; RR = 0.64; 95% CI 0.43–0.95), receiving an irregular frequency of social assistance payments (compared to receiving them regularly; RR = 1.74; 95% CI 1.31–2.31), having a prior offence (any conviction) during the 2-year period preceding the study period (RR = 3.60; 95% CI 2.65–4.90), drug dependence (RR = 1.47; 95% CI 1.08–2.01), and psychiatric (NSMD-related) hospitalization (RR = 1.49; 95% CI 1.06–2.08). The sensitivity analysis results were similar to the initial multivariable model. Results of the supplemental analysis involving the hierarchical GEE negative binomial regression model are presented in Supplementary Table S6; findings were similar to the initial model with none of the mental disorders being significantly associated with criminal convictions prior to or following adjustment for psychiatric hospitalization.

**Table 3:** GEE negative binomial regression analysis to identify risk and protective factors associated with the number of convicted offences (measured annually) among Vancouver At Home participants during the five years preceding study baseline (n = 425).

Variable	Unadjusted RR	*P* value	Adjusted RR	*P* value
	(95% CI)		(95% CI)	
Age (per year)	0.98 (0.97, 0.99)	**0.005**	0.98 (0.96, 1.00)	**0.015**
Time (per year)	1.14 (1.05, 1.24)	**0.001**	1.18 (1.08, 1.28)	** < 0.001**
Man	1.66 (1.10, 2.50)	**0.015**	1.49 (1.00, 2.20)	**0.047**
Indigenous	2.07 (1.37, 3.13)	**0.001**	1.36 (0.89, 2.09)	0.159
White	0.84 (0.59, 1.21)	0.349	0.98 (0.66, 1.45)	0.919
Education (less than high school)	1.98 (1.38, 2.82)	** < 0.001**	1.14 (0.82, 1.58)	0.433
Age of first homelessness (< 25 years)	1.40 (0.98, 2.01)	0.065	0.95 (0.67, 1.34)	0.756
Social assistance payments (yearly)				
No (0–1)	0.40 (0.20, 0.58)	** < 0.001**	0.64 (0.43, 0.95)	**0.028**
Irregular (2–11)	1.43 (1.09, 1.87)	**0.010**	1.74 (1.31, 2.31)	** < 0.001**
Regular (> 11)	Reference		Reference	
Prior offence (any conviction) during the 2-year period preceding study period (yes vs. no)^a^	4.55 (3.30, 6.26)	** < 0.001**	3.60 (2.65, 4.90)	** < 0.001**
Schizophrenia (yearly, yes vs. no)^a^	1.53 (1.10, 2.12)	**0.011**	1.04 (0.76, 1.41)	0.819
Bipolar disorder (yearly, yes vs. no)^a^	1.21 (0.89, 1.64)	0.218	0.89 (0.65, 1.21)	0.450
Neurotic disorder (yearly, yes vs. no) ^a^	1.29 (1.00, 1.66)	0.053	1.03 (0.76, 1.41)	0.842
Depressive disorder (yearly, yes vs. no)^a^	1.23 (0.95, 1.61)	0.121	0.91 (0.66, 1.25)	0.552
Personality disorder (yearly, yes vs. no)^a^	1.50 (1.14, 1.96)	**0.003**	1.16 (0.81, 1.66)	0.412
Alcohol dependence (yearly, yes vs. no)^a^	1.25 (0.80, 1.95)	0.332	0.98 (0.57, 1.69)	0.953
Drug dependence (yearly, yes vs. no)^a^	1.95 (1.43, 2.65)	** < 0.001**	1.47 (1.08, 2.01)	**0.015**
Nondependent drug abuse (yearly, yes vs. no)^a^	1.77 (1.21, 2.60)	** < 0.001**	1.25 (0.85, 1.84)	0.260
Psychiatric (NSMD-related) hospitalization (yearly, yes vs. no) ^a^	1.60 (1.22, 2.08)	**0.001**	1.49 (1.06, 2.08)	**0.021**
Substance use disorder-related hospitalization (yearly, yes vs. no)^a^	1.55 (1.12, 2.16)	** < 0.001**	1.31 (0.93, 1.85)	0.125
Non-psychiatric hospitalization (yearly, yes vs. no)^a^	1.37 (1.04, 1.81)	**0.025**	1.10 (0.80, 1.52)	0.548

^a^The reference group is “no”.

## Discussion

Our results emphasize prior convictions, irregular receipt of social assistance payments, drug dependence, psychiatric hospitalization, time, younger age, and being a man as factors associated with a greater rate of criminal convictions among PEHSMI. Some of the variables that have been found to be significantly associated with CJSI in prior studies using unadjusted or adjusted analyses among PEHSMI, such as age of first homelessness^44^, personality disorder^45^, or ethnicity^16^, were significant only in our unadjusted analyses and were no longer significant when included in our multivariable model. Furthermore, none of the serious mental disorders were significantly associated with convicted offences in our multivariable analyses, which is more consistent with the General Personality and Cognitive Social Learning model. However, psychiatric hospitalization was a significant risk factor for criminal convictions and may have accounted for the most symptomatic people experiencing serious mental illness. Taken collectively, the significant variables associated with convicted offences in our multivariable analysis suggest the need for publicly funded, long-term recovery-oriented support services that simultaneously address poverty, social marginalization, and mental health and substance use treatment needs, as the potential focus of intervention. Since abstinence from substance use may not be a part of recovery for many^46^, a range of recovery-oriented services should be available (i.e., non-abstinent and abstinent-contingent).

Consistent with previous research examining CJSI correlates among PEHSMI^16,44^, men had a significantly higher rate of criminal convictions. Older age was also protective against criminal convictions, which is consistent with prior studies using a variety of CJSI variables among PEHSMI and more generally among people involved in the criminal justice system^27,44,47,48^. Time was strongly associated with offending, consistent with evidence that an accumulation of time homeless increases the risk for CJSI among PEHSMI^12,44,45,47^. Maturation as a protective factor may be working in the opposite direction of exposure to homelessness, a structural risk factor. Increased recognition in the community, including by police, may have increased the likelihood of detention, arrest, and prosecution. It has also been argued that more time spent homeless may increase the frequency of committing crimes as an adaptive survival strategy^2^. Additionally, most participants of VAH were recruited from a neighbourhood in Vancouver called the Downtown Eastside^49^. A prior analysis demonstrated that VAH participants had increasingly migrated to the Downtown Eastside in the ten-year period preceding study baseline, accompanied by substantial increases in criminal convictions^49^. The finding of time being significantly associated with criminal convictions in the present study is consistent with those results.

Having a prior conviction before the study period was significantly associated with subsequent convictions during the study period, increasing the risk by 3.6 times compared to those without a prior conviction. Prior CJSI as a factor that increases the risk of future CJSI is a well-established finding not only among PEHSMI but also in other populations^8,26,27,48^ and is one of the Central Eight risk factors^25,26^.

Receiving an irregular frequency of social assistance payments was associated with a 74% higher rate of criminal convictions compared to receiving payments regularly. A similar finding was reported by McGuire and Rosenheck ^45^ but the relationship reported was unadjusted and involved lifetime incarceration (presence and duration) as the CJSI outcome. An irregular social assistance payment frequency was not only highly significant in the present study’s multivariable model but also had one of the largest effect sizes. This finding suggests that social assistance payment regularity serves to protect PEHSMI from resorting to crime. The receipt of no social assistance payments (0–1 payment) was significantly associated with a lower rate of convictions compared to those receiving payments regularly. Participants not receiving social assistance payments may have been employed or had incomes exceeding social assistance eligibility requirements. Employment is also one of the risk factors of the Central Eight. Policies facilitating social assistance payment consistency and removing administrative barriers to enrollment may contribute to reductions in CJSI. Furthermore, it may be that strengthening income assistance programs to enable recipients to cover basic subsistence needs may have the added benefit of reducing CJSI. Supported employment is an evidence-based intervention among people experiencing serious mental illness^50–52^, provision of which to people interested in becoming employed may have the added benefit of contributing to reductions in CJSI.

The presence of drug dependence was significantly associated with a 47% higher rate of convictions. Indeed, substance use-related variables have consistently been found to increase CJSI among PEHSMI^12,44,45,47^ and in other populations of people experiencing mental illness^26,27^. Substance abuse is also one of the Central Eight risk factors^25,26^. Drug dependence is the closest approximation to addiction in our multivariable model. The association between psychosocial marginalization and addictive use of drugs is well-established historically^53^. Participants of VAH had long histories of marginalization, including homelessness and unmet basic needs (e.g., food insecurity)^54^. Individual-level substance use treatment programs are important in facilitating recovery from addiction (e.g., contingency management, motivational interviewing, opioid agonist treatment, etc.), but the scope of such recovery includes more than substance use itself^46^. Services should also address and rectify structural factors that facilitate marginalization (e.g., homelessness) and hinder recovery from addictions. These services should be person- and family-centred as addiction recovery is individually defined^55^.

Lastly, hospitalization attributed to NSMD was significantly and independently associated with an increased risk of criminal convictions, while other types of hospitalization were not. This finding implicates the importance of symptom severity and may provide support for the criminalization of mentally disordered behaviour hypothesis. It may also represent people who were in crisis and who were highly distressed and symptomatic in response to the conditions of homelessness experienced. Indeed, homelessness duration has been found to significantly predict distress and may lead to a worsening of mental health symptoms^56^. Housing combined with health and social supports, including mental health treatment, may reduce hospitalization^57^. One study found that Housing First was associated with a reduction in psychiatric hospitalization days^58^. Using administrative data, Russolillo et al.^59^ reported significantly reduced emergency department use caused by the implementation of high-fidelity, choice-based Housing First. It is also possible, however, that some participants may need more structured and intensive services^23,60^.

On the other hand, the relationship between psychiatric hospitalization and criminal convictions may not necessarily implicate mental health symptoms as an explanation. For example, in a study of crime among PEHSMI, Fischer et al.^14^ found that recruitment of participants from psychiatric hospitals (versus the streets) was associated with a significantly higher risk of non-violent and violent crime. This finding remained significant despite having controlled for the effect of mental health symptom severity. The authors interpreted this finding as possibly suggesting that participants had initially been taken to hospital for committing an offence and continued offending post-discharge for reasons other than mental health symptoms.

Breach offences were the second most common type of offence committed by participants in the present study, and, as Roy et al.^44^ argue, logistical challenges, competing demands on the time of people experiencing homelessness and mental illness, as well as lack of understanding of court ordered conditions, may preclude adherence to such conditions. It could also be that those in crisis and who were highly symptomatic may have been less likely to adhere to court orders. A criminal justice system that is more sensitive to such structural constraints may lead to a reduction in involvement of PEHSMI, but further research is needed to confirm this. Moreover, Louden et al.^61^ found that probation officers may not only assess people on probation experiencing mental illness as being at higher risk of recidivism than people on probation without mental illness (even when both have the same risk as per structured risk assessment instruments), but that they may also select “more punitive responses” to breaches of probation conditions made by people experiencing mental illness versus people without mental illness (i.e., submitting a formal sanction rather than increased supervision and addressing the cause of the breach to prevent further occurrences). The authors suggested further provision of training for probation officers in effective strategies to address breaches of probation conditions and on the potential negative impacts of decision-making bias. Another challenge that needs to be addressed is the deficit of services specifically for people experiencing mental illness while on parole^62^. Compared to people on parole without mental health problems, those with mental health problems have a higher risk of recidivism (specifically parole revocation). And in the interest of safety, parole officers may choose reincarceration as a response to breaches of parole conditions in the absence of effective community-based mental health treatment options^62^.

Nearly six out of ten participants had at least one recorded offence in the ten years preceding study baseline. This prevalence rate is within the range of lifetime rates of convictions found among PEHSMI that were reported in a previous systematic review (28.1–80%)^8^. Our prevalence is likely an underestimate because convictions that occurred among participants prior to age 18 during the 10 years before baseline would not be included. Additionally, we found the vast majority of offences committed by participants were non-violent (79.7%) and instead mostly related to property, breaches of court orders, and crimes associated with drugs and alcohol. These findings are consistent with observations made by other researchers describing people experiencing homelessness or PEHSMI as more likely to have CJSI due to minor or non-violent crimes directly related to poverty and homelessness itself (e.g., visibility, survival, and subsistence needs)^1,2,14–16^.

### Limitations and implications for future research

A few limitations inherent to the present study should be noted. First, criminal convictions are just one type of CJSI; other types, such as arrests, were not available in the IMRI. Therefore, the present study likely underestimated the extent of CJSI among participants. Moreover, it may be that the risk and protective factors of other CJSI outcomes, such as arrests, may differ from those of criminal convictions among PEHSMI. It would be informative for future research to longitudinally investigate the factors associated with CJSI outcomes other than criminal convictions, using administrative data. Second, criminal convictions received by participants while under the age of 18 were not available in the IMRI. We included all other available data for participants while they were under the age of 18 during the study period (e.g., data used from the Ministries of Health and Social Development and Social Innovation). Although the 10-year rate and prevalence of criminal convictions may have been underestimated as a result of this limitation, we do not think results of the bivariate and multivariable analyses were considerably affected, as only 5.9% of participants did not have complete information regarding convictions during the study period due to the age restriction of the IMRI. Nevertheless, future research examining rates and risk factors for CJSI among PEHSMI should consider linking juvenile and adult criminal justice records for completeness, where permitted. Third, the service use of participants outside of BC would not have been captured in the IMRI, so all variables other than the self-reported ones collected at study baseline were potentially underestimated. This limitation would have likely affected the 10-year estimates of the rate and prevalence of criminal convictions more than the 5-year estimates and inferential statistics. This is because prior analyses of VAH data using the IMRI have shown that, at most, about 12% of participants lived outside of BC 5 years before study baseline, and this proportion decreased to 3% in the year before baseline. On the other hand, at most, 20% of participants lived outside of BC 10 years prior to baseline^49^. To overcome this limitation, national administrative databases may be utilized, where permitted and in existence. Fourth, participants had access to universal health insurance, and this may hinder generalizability to locales with different health insurance schemes. For example, in regions without universal health insurance, health services may be accessed less frequently or not at all. Future studies longitudinally investigating predictors of CJSI among PEHSMI using comprehensive administrative data in areas without universal health insurance can clarify how risk and protective factors may differ from areas with universal health insurance. Fifth, about 14.5% of participants in VAH were excluded from analyses because they did not provide consent to access their administrative data from all three ministries or could not be linked; any unmeasured differences between these participants and the ones included in analyses may affect results. However, as mentioned above, prior comparisons between VAH participants who provided consent and those who did not have shown no significant differences^42^. Sixth, any coding errors in the IMRI may have influenced results in any direction. This is a universal limitation of administrative database sources, and it is difficult to assess the extent of such coding errors and their impact on results. Seventh, due to lack of availability in the IMRI, additional covariates that have been found to be significantly associated with CJSI among PEHSMI could not be included in the present analyses, such as victimization^16,44^, mental health symptoms ^45,47^, childhood conduct disorder^12,45^, and homelessness duration^12,44,45,47^ in each year of the study period. These variables may have been significantly associated with a higher rate of criminal convictions if they were available and included in the present study’s bivariate and multivariable analyses. Moreover, in some cases, we compared constructs that have been measured differently in previous studies. More specifically, we used psychiatric hospitalization in place of mental health symptom severity^14^ and neurotic disorder in place of post-traumatic stress disorder^44^. Although psychiatric hospitalization may include people experiencing severe mental health symptoms, it is not a complete proxy for such symptoms as some people may not go to a hospital, and, as mentioned above, the significance of psychiatric hospitalization in multivariable analyses may not necessarily implicate mental health symptoms as an explanation^14^. Furthermore, as a limitation of the IMRI, emergency department visits from the National Ambulatory Care Reporting System were not available to be combined with psychiatric hospitalization, potentially resulting in an underestimation of participants experiencing severe mental health symptoms. Additionally, neurotic disorders include mental disorders other than post-traumatic stress disorder. Therefore, it will be important for future longitudinal research to include direct administrative measures of mental health symptoms (e.g., chart reviews) and diagnoses of post-traumatic stress disorder in multivariable analyses to clarify their relationship with CJSI among PEHMSI. Lastly, only “woman” and “man” were included as levels of the variable “gender” as few people self-identified as any other gender, limiting adequate statistical power to include additional genders in analyses. It may be necessary for future research to engage in statistical oversampling of people self-identifying as genders other than “woman” and “man” to be able to conduct inferential analyses on additional genders. This is particularly important given the paucity of research including gender beyond a binary approach in studies investigating risk factors for CJSI among PEHSMI.

## Conclusions

The findings of the present study highlight several variables in explaining the disproportionate CJSI of PEHSMI, as measured by criminal convictions. The importance of the present study’s results is that they show that some variables reported in previous studies are only significant in bivariate modelling. When multivariable modelling is employed using a comprehensive set of linked administrative data spanning multiple years, the overall pattern of results implicates poverty, social marginalization, crises involving mental illness, substance dependence, and the need for long-term recovery-oriented services that address these conditions. At the present time, Pathways Housing First offers the most comprehensive and evidence-based response that addresses each of these domains^63^.

## Supplementary Information

Supplementary Information.

## Data Availability

Data for the present analyses are available via e-mail request to J.M.S. (last author) at jsomers@sfu.ca.

## References

[1] Tsai J, Rosenheck RA, Kasprow WJ, McGuire JF (2014). Homelessness in a national sample of incarcerated veterans in state and federal prisons. Adm. Policy Ment. Health.

[2] Snow DA, Baker SG, Anderson L (1989). Criminality and homeless men: An empirical assessment. Soc. Probl..

[3] Gonzalez JR (2018). Criminal justice system involvement among homeless adults. Am. J. Crim. Justice.

[4] Gulati G (2019). The prevalence of major mental illness, substance misuse and homelessness in Irish prisoners: Systematic review and meta-analyses. Ir. J. Psychol. Med..

[5] Fazel S, Hayes AJ, Bartellas K, Clerici M, Trestman R (2016). The mental health of prisoners: A review of prevalence, adverse outcomes and interventions. Lancet Psychiatr..

[6] Baranyi G (2019). Severe mental illness and substance use disorders in prisoners in low-income and middle-income countries: A systematic review and meta-analysis of prevalence studies. Lancet Glob. Health.

[7] Prins SJ (2014). The prevalence of mental illnesses in U.S. state prisons: A systematic review. Psychiatr. Serv..

[8] Roy L, Crocker AG, Nicholls TL, Latimer EA, Ayllon AR (2014). Criminal behavior and victimization among homeless individuals with severe mental illness: A systematic review. Psychiatr. Serv..

[9] Greenberg GA, Rosenheck RA (2014). Psychiatric correlates of past incarceration in the national co-morbidity study replication. Crim. Behav. Ment. Health.

[10] Metraux S, Culhane DP (2004). Homeless shelter use and reincarceration following prison release. Criminol. Public Policy.

[11] Whittaker E (2015). Multiply disadvantaged: Health and service utilisation factors faced by homeless injecting drug consumers in Australia. Drug Alcohol Rev..

[12] Desai RA, Lam J, Rosenheck RA (2000). Childhood risk factors for criminal justice involvement in a sample of homeless people with serious mental illness. J. Nerv. Ment. Dis..

[13] Robinson T (2017). No right to rest: police enforcement patterns and quality of life consequences of the criminalization of homelessness. Urban Aff. Rev..

[14] Fischer SN, Shinn M, Shrout P, Tsemberis S (2008). Homelessness, mental illness, and criminal activity: Examining patterns over time. Am. J. Commun. Psychol..

[15] Kouyoumdjian FG (2019). Interactions between police and persons who experience homelessness and mental illness in Toronto, Canada: Findings from a prospective study. Can. J. Psychiatr..

[16] Roy L, Crocker AG, Nicholls TL, Latimer E, Isaak CA (2016). Predictors of criminal justice system trajectories of homeless adults living with mental illness. Int. J. Law Psychiatr..

[17] Fazel S, Wolf A, Palm C, Lichtenstein P (2014). Violent crime, suicide, and premature mortality in patients with schizophrenia and related disorders: A 38-year total population study in Sweden. Lancet Psychiatr..

[18] Fazel S (2015). Depression and violence: A Swedish population study. Lancet Psychiatr..

[19] Prins SJ (2011). Does transinstitutionalization explain the overrepresentation of people with serious mental illnesses in the criminal justice system?. Commun. Ment. Health J..

[20] Draine J, Salzer MS, Culhane DP, Hadley TR (2002). Role of social disadvantage in crime, joblessness, and homelessness among persons with serious mental illness. Psychiatr. Serv..

[21] Peterson J, Skeem JL, Hart E, Vidal S, Keith F (2010). Analyzing offense patterns as a function of mental illness to test the criminalization hypothesis. Psychiatr. Serv..

[22] Abramson MF (1972). The criminalization of mentally disordered behavior: Possible side-effect of a new mental health law. Hosp. Commun. Psychiatr..

[23] Lamb HR, Weinberger LE (2020). Deinstitutionalization and other factors in the criminalization of persons with serious mental illness and how it is being addressed. CNS Spectr..

[24] Lamberti JS, Katsetos V, Jacobowitz DB, Weisman RL (2020). Psychosis, mania and criminal recidivism: associations and implications for prevention. Harv. Rev. Psychiatr..

[25] Andrews DA, Bonta J (2010). The Psychology of Criminal Conduct.

[26] Bonta J, Blais J, Wilson HA (2014). A theoretically informed meta-analysis of the risk for general and violent recidivism for mentally disordered offenders. Aggress. Violent Behav..

[27] Bonta J, Law M, Hanson K (1998). The prediction of criminal and violent recidivism among mentally disordered offenders: a meta-analysis. Psychol. Bull..

[28] Lemieux AJ, Roy L, Martin MS, Latimer EA, Crocker AG (2017). Justice involvement among homeless individuals with mental illnesses: Are self-report and administrative measures comparable?. Eval. Program Plann..

[29] Somers JM (2016). Accuracy of reported service use in a cohort of people who are chronically homeless and seriously mentally ill. BMC Psychiatr..

[30] Somers JM (2013). Vancouver At Home: pragmatic randomized trials investigating Housing First for homeless and mentally ill adults. Trials.

[31] Sheehan DV (1998). The Mini-International Neuropsychiatric Interview (M.I.N.I.): The development and validation of a structured diagnostic psychiatric interview for DSM-IV and ICD-10. J. Clin. Psychiatr..

[32] Barker S, Barron N, McFarland BH, Bigelow DA (1994). A community ability scale for chronically mentally ill consumers: part I. Reliability and validity. Commun. Ment. Health J..

[33] Barker S, Barron N, McFarland BH, Bigelow DA, Carnahan T (1994). A community ability scale for chronically mentally ill consumers: Part II. Applications. Commun. Ment. Health J..

[34] Somers JM, Moniruzzaman A, Rezansoff SN, Brink J, Russolillo A (2016). The prevalence and geographic distribution of complex co-occurring disorders: A population study. Epidemiol. Psychiatr. Sci..

[35] Fazel S, Zetterqvist J, Larsson H, Långström N, Lichtenstein P (2014). Antipsychotics, mood stabilisers, and risk of violent crime. Lancet.

[36] Rezansoff SN, Moniruzzaman A, Fazel S, McCandless L, Somers JM (2017). Adherence to antipsychotic medication and criminal recidivism in a Canadian provincial offender population. Schizophr. Bull..

[37] Zeger SL, Liang K-Y (1986). Longitudinal data analysis for discrete and continuous outcomes. Biometrics.

[38] White H (1980). A heteroskedasticity-consistent covariance matrix estimator and a direct test for heteroskedasticity. Econometrica.

[39] Hilbe, J. M. *Negative Binomial Regression* 2nd edn. (Cambridge University Press, 2011).

[40] Hilbe, J. M. *Modeling Count Data*. (Cambridge University Press, 2014).

[41] StataCorp. Stata Statistical Software (Release 16). StataCorp LLC. (2019).

[42] Rezansoff SN, Moniruzzaman A, Fazel S, Procyshyn R, Somers JM (2016). Adherence to antipsychotic medication among homeless adults in Vancouver, Canada: A 15-year retrospective cohort study. Soc. Psychiatr. Psychiatr. Epidemiol..

[43] Russolillo A, Moniruzzaman A, Parpouchi M, Currie LB, Somers JM (2016). A 10-year retrospective analysis of hospital admissions and length of stay among a cohort of homeless adults in Vancouver, Canada. BMC Health Serv. Res..

[44] Roy L (2016). Profiles of criminal justice system involvement of mentally ill homeless adults. Int. J. Law Psychiatr..

[45] McGuire JF, Rosenheck RA (2004). Criminal history as a prognostic indicator in the treatment of homeless people with severe mental illness. Psychiatr. Serv..

[46] Martinelli TF (2020). Comparing three stages of addiction recovery: Long-term recovery and its relation to housing problems, crime, occupation situation, and substance use. Drugs Educ. Prev. Policy.

[47] Calsyn RJ, Yonker RD, Lemming MR, Morse GA, Klinkenberg WD (2005). Impact of assertive community treatment and client characteristics on criminal justice outcomes in dual disorder homeless individuals. Crim. Behav. Ment. Health.

[48] Rezansoff SN, Moniruzzaman A, Gress C, Somers JM (2013). Psychiatric diagnoses and multiyear criminal recidivism in a Canadian provincial offender population. Psychol. Public Policy Law.

[49] Somers JM, Moniruzzaman A, Rezansoff SN (2016). Migration to the Downtown Eastside neighbourhood of Vancouver and changes in service use in a cohort of mentally ill homeless adults: A 10-year retrospective study. BMJ Open.

[50] Frederick DE, VanderWeele TJ (2019). Supported employment: meta-analysis and review of randomized controlled trials of individual placement and support. PLoS ONE.

[51] Richter D, Hoffmann H (2019). Effectiveness of supported employment in non-trial routine implementation: Systematic review and meta-analysis. Soc. Psychiatry Psychiatr. Epidemiol..

[52] Suijkerbuijk YB (2017). Interventions for obtaining and maintaining employment in adults with severe mental illness, a network meta-analysis. Cochrane Database Syst. Rev..

[53] Alexander, B. *The Globalisation of Addiction: A Study in Poverty of the Spirit*. (Oxford University Press, 2008).

[54] Parpouchi M, Moniruzzaman A, Russolillo A, Somers JM (2016). Food insecurity among homeless adults with mental illness. PLoS ONE.

[55] Davidson L, White W (2007). The concept of recovery as an organizing principle for integrating mental health and addiction services. J. Behav. Heal. Serv. Res..

[56] Castellow J, Kloos B, Townley G (2015). Previous homelessness as a risk factor for recovery from serious mental illnesses. Community Ment. Health J..

[57] Culhane DP, Metraux S, Hadley T (2002). Public service reductions associated with placement of homeless persons with severe mental illness in supportive housing. Hous. Policy Debate.

[58] Gulcur L, Stefancic A, Shinn M, Tsemberis S, Fischer SN (2003). Housing, hospitalization, and cost outcomes for homeless individuals with psychiatric disabilities participating in continuum of care and housing first programmes. J. Commun. Appl. Soc. Psychol..

[59] Russolillo A, Patterson M, McCandless L, Moniruzzaman A, Somers J (2014). Emergency department utilisation among formerly homeless adults with mental disorders after one year of Housing First interventions: a randomised controlled trial. Int. J. Hous. Policy.

[60] Lamb HR, Weinberger LE (2017). Understanding and treating offenders with serious mental illness in public sector mental health. Behav. Sci. Law.

[61] Eno LJ, Mancha SM, Ricks EP, Kennealy PJ (2018). The role of stigma toward mental illness in probation officers’ perceptions of risk and case management decisions. Crim. Justice Behav..

[62] Ostermann M, Matejkowski J (2014). Exploring the intersection of mental health and release status with recidivism. Justice Q..

[63] Tsemberis, S. *Housing First: The Pathways Model to End Homelessness for People With Mental Health and Substance Use Disorders*. (Hazelden Publishing, 2015).

