# Supplementary material for: Multivariable modelling of factors associated with criminal convictions among people experiencing homelessness and serious mental illness: a multi-year study
Source: Sci Rep. 2021 Aug 16;11:16610. doi: 10.1038/s41598-021-96186-x (PMC8368183; doi:10.1038/s41598-021-96186-x)
Supplement: Supplementary file 1 — Supplementary Information. [file 41598_2021_96186_MOESM1_ESM.pdf]

Multivariable Modelling of Factors Associated With Criminal Convictions Among People Experiencing Homelessness and Serious Mental Illness: A Multi-Year Study

Milad Parpouchi, Akm Moniruzzaman, Jane A. Buxton, Julian M. Somers

**ICD-9[a] Code**	**Description**
290	Senile and presenile organic psychotic conditions
291[b]	*Alcoholic psychoses*
292[b]	*Drug psychoses*
293	Transient organic psychotic conditions
294	Other organic psychotic conditions (chronic)
295	Schizophrenic psychoses
296	Affective psychoses
297	Paranoid states
298	Other nonorganic psychoses
299	Psychoses with origin specific to childhood
300	Neurotic disorders
301	Personality disorders
302	Sexual deviations and disorders
303[b]	*Alcohol dependence syndrome*
304[b]	*Drug dependence*
305[b]	*Nondependent abuse of drugs*
306	Physiological malfunction arising from mental factors
307	Special symptoms or syndromes not elsewhere classified
308	Acute reaction to stress
309	Adjustment reaction
310	Specific nonpsychotic mental disorders following organic brain damage
311	Depressive disorder, not elsewhere classified
312	Disturbance of conduct not elsewhere classified
313	Disturbance of emotions specific to childhood and adolescence
314	Hyperkinetic syndrome of childhood
315	Specific delays in development
316	Psychic factors associated with diseases classified elsewhere
317	Mild mental retardation
318	Other specified mental retardation
319	Unspecified mental retardation
50B	Anxiety/depression

Supplementary Table S1. ICD-9 diagnostic codes (290-319; mental disorders). This table includes ICD-9 codes and descriptions of mental disorders from the document titled "Mental disorders" by the British Columbia Medical Services Plan, accessed on May 18, 2016. URL: http://www2.gov.bc.ca/assets/gov/health/practitioner-pro/medical-services-plan/diag-codes_mental.pdf.
[a]ICD-9: The International Classification of Diseases, Ninth Revision. [b]Diagnostic codes used for substance use disorders.

ICD-10-CA[a] Code	Description of disorder
F00-F09	Organic, including symptomatic mental disorders
F10-F19	Mental & behavioural disorders due to psychoactive substance use
F20-F29	Schizophrenia, schizotypal & delusional disorders
F30-F39	Mood [affective] disorders
F40-F48	Neurotic, stress related & somatoform disorders
F50-F59	Behavioural syndromes associated with psychological disturbances & physical factors
F60-F69	Disorders of adult personality & behaviour
F70-F79	Mental retardation
F80-F89	Disorders of psychological development
F90-F98	Behavioural and emotional disorders with onset usually occurring in childhood and adolescence
F99	Unspecified mental disorder

Supplementary Table S2. ICD-10-CA diagnostic codes (F00-F99; mental and behavioural disorders). This table includes ICD-10-CA codes and descriptions of mental disorders from the document titled "International Statistical Classification of Diseases and Related Health Problems, Tenth Revision, Canada (ICD-10-CA), Volume 1 -Tabular List (ISBN 1-55392-804-0)" by the Canadian Institute for Health Information ©2009. [a]ICD-10-CA: The International Classification of Diseases, Tenth Revision, Canada.

Mental/substance use disorder	ICD-9[a] code	Description of disorder
Schizophrenia	295	Schizophrenic psychoses
Bipolar disorder	296	Affective psychoses
Neurotic disorder	300	Neurotic disorders
Personality disorder	301	Personality disorders
Depressive disorder	311	Depressive disorder, not elsewhere specified
Alcohol dependence	303	Alcohol dependence syndrome
Drug dependence	304	Drug dependence
Nondependent drug abuse	305	Nondependent abuse of drugs

Supplementary Table S3. ICD-9 diagnostic codes and descriptions of mental and substance use disorders included in analyses. This table includes ICD-9 codes and descriptions of mental disorders from the document titled "Mental disorders" by the British Columbia Medical Services Plan, accessed on May 18, 2016. URL: http://www2.gov.bc.ca/assets/gov/health/practitioner-pro/medical-services-plan/diag-codes_mental.pdf. [a]ICD-9: The International Classification of Diseases, Ninth Revision.

Type of hospitalization	ICD-10-CA[a] code	Description of disorders
Psychiatric (NSMD[b]-related)	F00-F99, except F10-F19	Shown in appendix 2
Substance use disorder-related	F10-F19	Shown in appendix 2
Nonpsychiatric	All codes except F00-F99	Shown in appendix 2

Supplementary Table S4. ICD-10-CA diagnostic codes and descriptions of mental and behavioural disorders used for hospitalizations included in analyses. This table includes ICD-10-CA codes and descriptions of mental disorders from the document titled "International Statistical Classification of Diseases and Related Health Problems, Tenth Revision, Canada (ICD-10-CA), Volume 1 -Tabular List (ISBN 1-55392-804-0)" by the Canadian Institute for Health Information ©2009. [a]ICD-10-CA: The

International Classification of Diseases, Tenth Revision, Canada. [b]NSMD: non-substance use-related mental disorder.

**Variable**	**All Vancouver At Home participants (n=497) Mean (SD)/ n (%)**	**Eligible participants[a] (n=433) Mean (SD)/ n (%)**	**Ineligible participants[b] (n=64) Mean (SD)/ n (%)**	**P value[c]**
Age at randomization (in years)<br>Mean (SD)	40.8 (11.0)	40.8 (11.0)	41.4 (11.0)	0.682
Gender (woman)	134 (27)	112 (26)	22 (34)	0.165
Ethnicity				
Indigenous	77 (16)	70 (16)	7 (11)	0.054
White	280 (56)	235 (54)	45 (70)	
Other	140 (28)	128 (30)	12 (19)	
Education (less than high school)	280 (57)	247 (57)	33 (52)	0.376
Age of first homelessness (in years)<br>Mean (SD)	30.3 (13.3)	30.1 (13.4)	31.9 (12.6)	0.301

Supplementary Table S5. Socio-demographic characteristics of participants of the Vancouver At Home study by administrative data status at study baseline. [a]433 participants consented to the research team accessing their administrative health records and could be linked. [b]60 participants did not consent to the research team accessing their administrative health records, and 4 provided consent but could not be linked. [c]P values are based on significance testing of differences in characteristics between eligible (n=433) and ineligible (n=64) participants.

Variable	Block 1 Adjusted RR (95% CI)[a]	Block 2 Adjusted RR (95% CI)[a]	Block 3 Adjusted RR (95% CI)[a]
Age (per year)	**0.98 (0.96, 0.99)**	**0.98 (0.96, 1.00)**	**0.98 (0.96, 1.00)**
Time (per year)	**1.22 (1.12, 1.32)**	**1.18 (1.09, 1.28)**	**1.18 (1.08, 1.28)**
Man	1.46 (0.97, 2.22)	**1.50 (1.01, 2.22)**	**1.49 (1.00, 2.20)**
Indigenous	1.36 (0.89, 2.07)	1.35 (0.88, 2.06)	1.36 (0.89, 2.09)
White	1.02 (0.68, 1.54)	0.95 (0.64, 1.41)	0.98 (0.66, 1.45)
Education (less than high school)	1.12 (0.81, 1.53)	1.14 (0.82, 1.57)	1.14 (0.82, 1.58)
Age of first homelessness (<25 years)	0.94 (0.68, 1.30)	0.95 (0.67, 1.34)	0.95 (0.67, 1.34)
Social assistance payments (yearly)			
No (0-1)	**0.54 (0.37, 0.80)**	**0.64 (0.42, 0.96)**	**0.64 (0.43, 0.95)**
Irregular (2-11)	**1.76 (1.33, 2.33)**	**1.76 (1.32, 2.33)**	**1.74 (1.31, 2.31)**
Regular (> 11)	Reference	Reference	Reference
Prior offence (any conviction) during the 2-year period preceding study period (yes vs. no)[b]	**3.81 (2.83, 5.13)**	**3.53 (2.60, 4.80)**	**3.60 (2.65, 4.90)**
Schizophrenia (yearly, yes vs. no)[b]		1.27 (0.93, 1.73)	1.04 (0.76, 1.41)
Bipolar disorder (yearly, yes vs. no)[b]		1.03 (0.73, 1.44)	0.89 (0.65, 1.21)
Neurotic disorder (yearly, yes vs. no)[b]		1.06 (0.78, 1.44)	1.03 (0.76, 1.41)
Depressive disorder (yearly, yes vs. no)[b]		0.94 (0.69, 1.28)	0.91 (0.66, 1.25)
Personality disorder (yearly, yes vs. no)[b]		1.24 (0.88, 1.74)	1.16 (0.81, 1.66)
Alcohol dependence (yearly, yes vs. no)[b]		1.06 (0.64, 1.77)	0.98 (0.57, 1.69)
Drug dependence (yearly, yes vs. no)[b]		**1.56 (1.14, 2.12)**	**1.47 (1.08, 2.01)**

Variable	Block 1 Adjusted RR (95% CI)[a]	Block 2 Adjusted RR (95% CI)[a]	Block 3 Adjusted RR (95% CI)[a]
Nondependent drug abuse (yearly, yes vs. no)[b]		1.37 (0.96, 1.95)	1.25 (0.85, 1.84)
Psychiatric (NSMD-related) hospitalization (yearly, yes vs. no)[b]			**1.49 (1.06, 2.08)**
Substance use disorder-related hospitalization (yearly, yes vs. no)[b]			1.31 (0.93, 1.85)
Non-psychiatric hospitalization (yearly, yes vs. no)[b]			1.10 (0.80, 1.52)

Supplementary Table S6. Hierarchical generalized estimating equations negative binomial regression analysis to identify risk and protective factors associated with the number of convicted offences (measured annually) among Vancouver At Home participants during the five years preceding study baseline (n=425). [a]Bold indicates significance at $p \leq 0.05$. [b]The reference group is “no”.
